# Mutation or Loss of p53 Differentially Modifies TGFβ Action in Ovarian Cancer

**DOI:** 10.1371/journal.pone.0089553

**Published:** 2014-02-20

**Authors:** Eoghainín Ó hAinmhire, Suzanne M. Quartuccio, Whay Cheng, Roshan A. Ahmed, Shelby M. King, Joanna E. Burdette

**Affiliations:** Department of Medicinal Chemistry and Pharmacognosy, Center for Pharmaceutical Biotechnology, University of Illinois at Chicago, Chicago, Illinois, United States of America; Virginia Commonwealth University, United States of America

## Abstract

Ovarian cancer is the most lethal gynecological disease affecting women in the US. The Cancer Genome Atlas Network identified p53 mutations in 96% of high-grade serous ovarian carcinomas, demonstrating its critical role. Additionally, the Transforming Growth Factor Beta (TGFβ) pathway is dysfunctional in various malignancies, including ovarian cancer. This study investigated how expression of wild-type, mutant, or the absence of p53 alters ovarian cancer cell response to TGFβ signaling, as well as the response of the ovarian surface epithelium and the fallopian tube epithelium to TGFβ. Only ovarian cancer cells expressing wild-type p53 were growth inhibited by TGFβ, while ovarian cancer cells that were mutant or null p53 were not. TGFβ induced migration in p53 null SKOV3 cells, which was not observed in SKOV3 cells with stable expression of mutant p53 R273H. Knockdown of wild-type p53 in the OVCA 420 ovarian cancer cells enhanced cell migration in response to TGFβ. Increased protein expression of DKK1 and TMEPAI, two pro-invasive genes with enhanced expression in late stage metastatic ovarian cancer, was observed in p53 knockdown and null cells, while cells stably expressing mutant p53 demonstrated lower DKK1 and TMEPAI induction. Expression of mutant p53 or loss of p53 permit continued proliferation of ovarian cancer cell lines in the presence of TGFβ; however, cells expressing mutant p53 exhibit reduced migration and decreased protein levels of DKK1 and TMEPAI.

## Introduction

Ovarian cancer is the fifth leading cause of cancer death and the most lethal gynecologic disease among US women. In 2013, an estimated 22,240 cases of ovarian cancer will be diagnosed, resulting in 14,030 deaths [1]. The high mortality rate can be attributed to the fact that over 60% of ovarian cancers will be diagnosed after the disease has spread to distant locations. Once metastasized, the five-year survival rate drops to under 30% [1]. Inefficiency of diagnosis is primarily due to a lack of understanding of the initiating events and mechanisms of progression that give rise to ovarian cancer, with few early detection strategies [2]. Importantly, if ovarian cancer is diagnosed earlier, survival rates can be as high as 90% [1]. These statistics illustrate the fundamental need to better understand early mechanistic events of ovarian cancer that will assist with earlier diagnosis and better prognosis of patients.

The tumor suppressor p53 is the most commonly mutated gene in all human cancers [2]. p53 is a transcription factor that controls many cellular functions such as the cell cycle, apoptosis, and response to DNA damage [3]. Most *TP53* mutations are missense mutations, where a single nucleotide base substitution results in either dysfunction or absence of p53 activity [4]. These mutations lead to increased proliferation, invasion, and metastasis in many cancers [5]. The Cancer Genome Atlas Network (CGAN) identified p53 as being mutated in up to 96% of chemotherapy resistant, high-grade serous ovarian cancers, indicating an essential role for p53 mutations in serous ovarian cancer [6]. Moreover, the International Agency for Research on Cancer (IARC) *TP53* database indicates that the most frequent p53 mutation in serous ovarian cancer is an arginine to histidine conversion at amino acid residue 273 (R273H) within the DNA binding domain, which accounts for 8% of all p53 mutations [7]. Mutant p53 R273H has been reported to play a role in promoting breast and lung cancer metastasis [8], [9] by increasing migration and invasion.

Another important signaling pathway that is modified in ovarian cancer is the Transforming Growth Factor Beta pathway (TGFβ) [10]. TGFβ is a superfamily of peptide growth factors that regulate growth, differentiation, apoptosis, and migration [10]. TGFβ signals by binding to a family of serine/threonine kinase membrane receptors, which phosphorylate downstream signaling molecules, primarily Smads 2 and 3 [11]. Once activated, these Smad complexes translocate to the nucleus and interact with various co-activators and repressors to modulate Smad-regulated transcription [11], [12]. TGFβ plays an important role in inducing growth arrest in normal ovarian cells [13]. In some cancer cells, TGFβ induces apoptosis and cell cycle arrest, while in other cancer cells it loses the ability to induce growth arrest and can instead promote cellular invasion [10]. It can also play a role in chemoresistance in advanced serous ovarian cancers [14]. The core TGFβ pathway components, the TGFβ receptors, and Smad proteins, are rarely mutated or lost in ovarian cancer [5], suggesting that disruption of the TGFβ pathway occurs by other mechanisms.

As p53 and Smads are both transcription factors, p53 is capable of interacting with Smads to modify both the p53 and TGFβ signaling pathways [4]. Smads can form a transcriptional complex with p53 to induce expression of genes that promote cell cycle arrest, such as p21 [4]. Smads and p53 bind to their own responsive elements in the promoters of TGFβ-responsive genes to synergistically activate or repress transcription [4]. In fact, it has been shown that p53 is required for TGFβ-induced cell cycle arrest [4]. Additionally, mutant p53 R273H abrogates TGFβ-induced cell cycle arrest and promotes metastatic behavior by blocking p63 in breast carcinoma cells [8]. In these cells, the mutant p53/Smad complex inhibits p63-mediated transcription leading to invasion in the presence of oncogenic Ras [8].

Given the high percentage of p53 mutations in ovarian tumors [6] and the recent evidence that p53 and Smads interact to regulate metastasis in breast carcinoma cells [8], the role of mutant p53 in response to TGFβ signaling in ovarian cancer was investigated. p53 and TGFβ are implicated in many cancers such as breast and lung on their own [15], [16] and in concert [8]. In breast and lung cancers, mutant p53 interacts with Smads to alter transcription of genes that regulate metastasis [8], but little is known about how p53 and TGFβ interact in ovarian cancer. Factors necessary for growth and metastasis in breast, lung, and colon cancer may not be necessary in ovarian cancer, leading to tissue specific effects of mutant p53 signaling [17]. Two genes (*TMEPAI* and *DKK1*) were studied based on their role in metastasis in ovarian cancer. *TMEPAI* is a TGFβ-induced negative regulator [18] and is involved in TGFβ-induced metastasis in breast carcinoma cell lines [18] but has never been reported to be co-regulated by p53 and Smads. *DKK1* is an inhibitor of Wnt signaling, which is often upregulated in metastatic ovarian cancer, and is associated with poor prognosis [19]. Maspin, an anti-metastatic protein, was also chosen as it has established regulation by p53 and Smads [20]. The current study evaluated whether expression of one of the most common p53 mutations in ovarian cancer (R273H) alters the cell response to Smad signaling to modulate cell proliferation and migration.

## Materials and Methods

### Cell culture

All reagents were obtained from Life Technologies (Carlsbad, CA) unless otherwise indicated. OVCA 420, OVCA 429, and OVCA 432 are cell lines that have been previously published [21], [22], while OVCAR5 cells are available through the national cancer institute (NCI) as part of the NCI60 tumor cell line anticancer drug screen [23]. The OVCA 420, OVCA 429, OVCA 432, and OVCAR5 cells (gifts from Dr. Gustavo Rodriguez and Dr. Teresa Woodruff at Northwestern University) were maintained in Minimum Essential Media (MEM) supplemented with 10% fetal bovine serum (FBS), 1% L-glutamine, 1% non-essential amino acids, 1% sodium pyruvate, and 1% penicillin/streptomycin. OVCAR3 cells were obtained from ATCC (Manassas, VA) and maintained in the same media as above, with the exception of supplementation with 20% FBS. SKOV3 cells were acquired from ATCC and maintained in McCoy's 5A supplemented with 2.3 g/L sodium carbonate, 10% FBS, and 1% penicillin/streptomycin.

Stable cell lines were selected using antibiotic resistant plasmids containing the gene of interest. SKOV3 cells stably expressing mutant p53 R273H [24] (Addgene, plasmid: 16439, donated by Dr. Vogelstein, Johns Hopkins University school of Medicine, Baltimore, MD) were selected using 500 μg/mL G418 (Gemini bio-products, West Sacramento, CA) and maintained in SKOV3 media containing 200 μg/mL G418. OVCA 420 cells expressing p53 shRNA or scrambled shRNA (Sigma-Aldrich, St. Louis, MO) were selected using 4 μg/mL puromycin (Sigma-Aldrich) and maintained with 1 μg/mL puromycin. p53 wild-type plasmid [24] was purchased from Addgene (Addgene plasmid: 16434, donated by Dr. Vogelstein, Johns Hopkins University school of Medicine, Baltimore, MD).

Normal immortalized human ovarian surface epithelial cells (IOSE 80) were a gift from Dr. Nelly Auersperg at the University of Vancouver and were maintained in 50% v/v Medium 199 and 50% v/v MCDB (Sigma-Aldrich, St. Louis, MO), 15% FBS, 1% L-glutamine, 1% penicillin/streptomycin, and 0.055% epithelial growth factor (EGF, PeproTech Inc, Rocky Hill, NJ) [25]. Normal human fallopian tube secretory epithelial cells (FTSEC) were a gift from Dr. Ronny Drapkin at Harvard University and were maintained in 50% v/v DMEM and 50% v/v F-12 (Mediatech, Manassas, VA), 1% L-glutamine, 1% penicillin/streptomycin, and 2% Ultroser G (Pall Corporation, Port Washington, NY) [26]. Mouse ovarian surface epithelial cells (MOSE) were isolated from C57BL/6 mice and mouse tubal epithelial cells (MTEC) were isolated from CD1 mice as previously described [27]. Cultured cells were maintained at 37°C in a 5% CO_2_ incubator.

### Luciferase assay

Cells were plated at a density of 25,000 per well into 24-well plates and incubated overnight. Cells were transfected with 0.05 μg/well of an expression construct containing the Smad binding element promoter upstream of the luciferase gene using Mirus TransIT LT1 (Mirus Bio LLC, Madison, WI) according to the manufacturer's instructions. The Smad responsive element plasmid contains a CAGA sequence repeated twelve times upstream of the luciferase gene (gift from Dr. Aris Moustakas at Ludwig Institute for Cancer Research, Uppsala, Sweden). Plasmids for expression of wild-type p53 or mutant p53 R273H plasmids were transfected into cells at 0.05 μg/mL. Cells were transfected for 24 hr in serum-supplemented media. Cells were then washed with PBS and treated with TGFβ1 at 10 ng/mL (Sigma Aldrich) for 24 hr. SB-431542 (Selleck Chemicals, Houston, TX) was used at a concentration of 5 μM for all luciferase assays. The protocol and SBE-luciferase transfection efficiencies were normalized and run as previously described [28]. Normal cell luciferase activity was measured using a Synergy Mx (BioTek, Winooski, VT).

### Proliferation assay

Sulforhodamine B (SRB) assays were used to determine cell density. Cells were treated for 48 hr with TGFβ (20 ng/mL) followed by colorimetric assay as previously described [29]. Cell survival was calculated by comparing the absorbance values between treated and control wells. Background was subtracted by measuring the absorbance of 0.1 mM of Tris-base alone.

### Flow cytometry

OVCA 420, OVCA 432, and SKOV3 cells were plated into T25 flasks 24 hr before treatment. Medium containing TGFβ (20 ng/mL) or solvent control was added and incubated for 48 hr. Following treatment, cells were trypsinized, washed with PBS, resuspended in 500 μL PBS, then fixed in 4 mL of 70% ethanol, and stored at −20°C overnight. The fixed cells were washed with PBS and stained with 500 μL propidium iodide (PI) solution [50 μg/mL PI, 90 units RNase A, 0.1% Triton X-100, 4 mmol/L citrate buffer, 10 mM polyethylene glycol (PEG) 4000]. Cells were incubated in the PI solution for 20 min at 37°C before being treated with 500 μL PI salt solution (1 mg/mL PI, 0.1 mL of 10% Triton X-100, 4 M NaCl solution, 10 mM PEG 4000). Flow cytometric analysis was done on a Beckman Coulter Elite ESP (Miami, FL) with at least 30,000 individual events per reaction. Data was analyzed with Mod-fit software (Verity Software House, Inc., Topsham, ME).

### Western blot analysis

Cells were plated at 50,000 cells per well in six-well plates, transfected with appropriate plasmids at 0.05 μg/mL, and treated with TGFβ1 (10 ng/mL) for 24 hr. To induce p53 express, cisplatin (Fisher Scientific, NC9343338) treatment was performed at 125 μM for two hours. Protein concentration was determined by BCA assay (Pierce, Rockford, IL). Cell lysate (30 μg) was analyzed by 10% SDS-PAGE and transferred to nitrocellulose. Blots were then blocked with 5% milk in TBS-T and probed overnight with primary antibodies. The antibodies used were human p53 (#9282), p21 (#9247), maspin (#9117), CDC2 (#9112) (Cell Signaling Technology, Inc., Beverly, MA) at a concentration of 1∶1000; DKK1 (H-120) and mouse p21 (F-5) (Santa Cruz Technology, Inc, Santa Cruz, CA) was used at a concentration of 1∶200; TMEPAI 2A12 (Abnova, Taipei, Tiawan) was used at a concentration of 1∶500; and actin (Sigma-Aldrich) at a concentration of 1∶1000. Anti-mouse and rabbit HRP-linked secondary (Cell Signaling Technology, Inc.) was used for all blots at a concentration of 1∶1000.

### Wound healing assay

Cells were plated at 50,000 cells per well in a 24-well plate and incubated overnight. A uniform wound was created through the cell monolayer using a pipette tip. Cells were washed and treated with TGFβ1 (20 ng/mL) immediately after scratching. Pictures were taken at 0, 24, and 48 hr after scratching, and the area of the scratch was analyzed with ImageJ software (National Institutes of Health, Bethesda, MD). Percent closure was measured compared to 0 hr and fold change was determined from percent closure of treated compared to untreated.

### Animals, organ culture and immunohistochemistry (IHC)

Animals were obtained, treated, and housed as previously described [27]. Ovaries and oviducts were dissected and cultured as previously described [30], [31]. The growth media consisted of alpha-MEM (Invitrogen), and 1% penicillin/streptomycin (Invitrogen) with 0.1% DMSO, 20 ng/μL TGFβ, 5 μM SB431542 (TGFβ inhibitor), or 20 ng/μL TGFβ plus 5 μM SB431542 added as treatment conditions. TGFβ was dissolved in water but 0.1% of DMSO was added to the TGFβ alone condition to control for SB431542 solvent. Bromodeoxyuridine (BrdU, Sigma; 10 μM) was added into the growth media 24 hr prior to tissue fixation. Tissues were prepared for paraffin sectioning and immunohistochemistry was completed as described previously [27].

### Proliferation imaging

Imaging was performed using a Nikon E600 Microscope with a DS-Ri1 Digital Camera and NIS Elements Software (Nikon Instruments, Melville, NY). ImageJ was used to quantify cell proliferation. Percent proliferation was calculated by dividing the number of epithelial cells staining positive for BrdU by the total number of epithelial cells.

### Ethics statement

All animals were treated in accordance with the National Institutes of Health Guidelines for the Care and Use of Laboratory Animals and the established Institutional Animal Use and Care protocol at the University of Illinois at Chicago. The protocol was approved by the Animal Care Committee at the University of Illinois at Chicago (protocol number: A08–250). Animals were housed in a temperature and light controlled environment (12 h light, 12 h dark) and were provided food and water ad libitum. All mice were euthanized by CO_2_ inhalation followed by cervical dislocation.

### Statistical analyses

All values are presented as the mean ± the standard error. ANOVA followed by Tukey's multiple comparison tests were used to assess differences between experimental and control groups. For the wound healing assay, a paired t-test was used to analyze control and treated in each cell line, while an unpaired t-test was used when comparing treated groups between two different cell lines. p<0.05 was considered statistically significant.

## Results

### TGFβ induces growth arrest in ovarian cancer cells expressing wild-type p53

**Figure 1:**
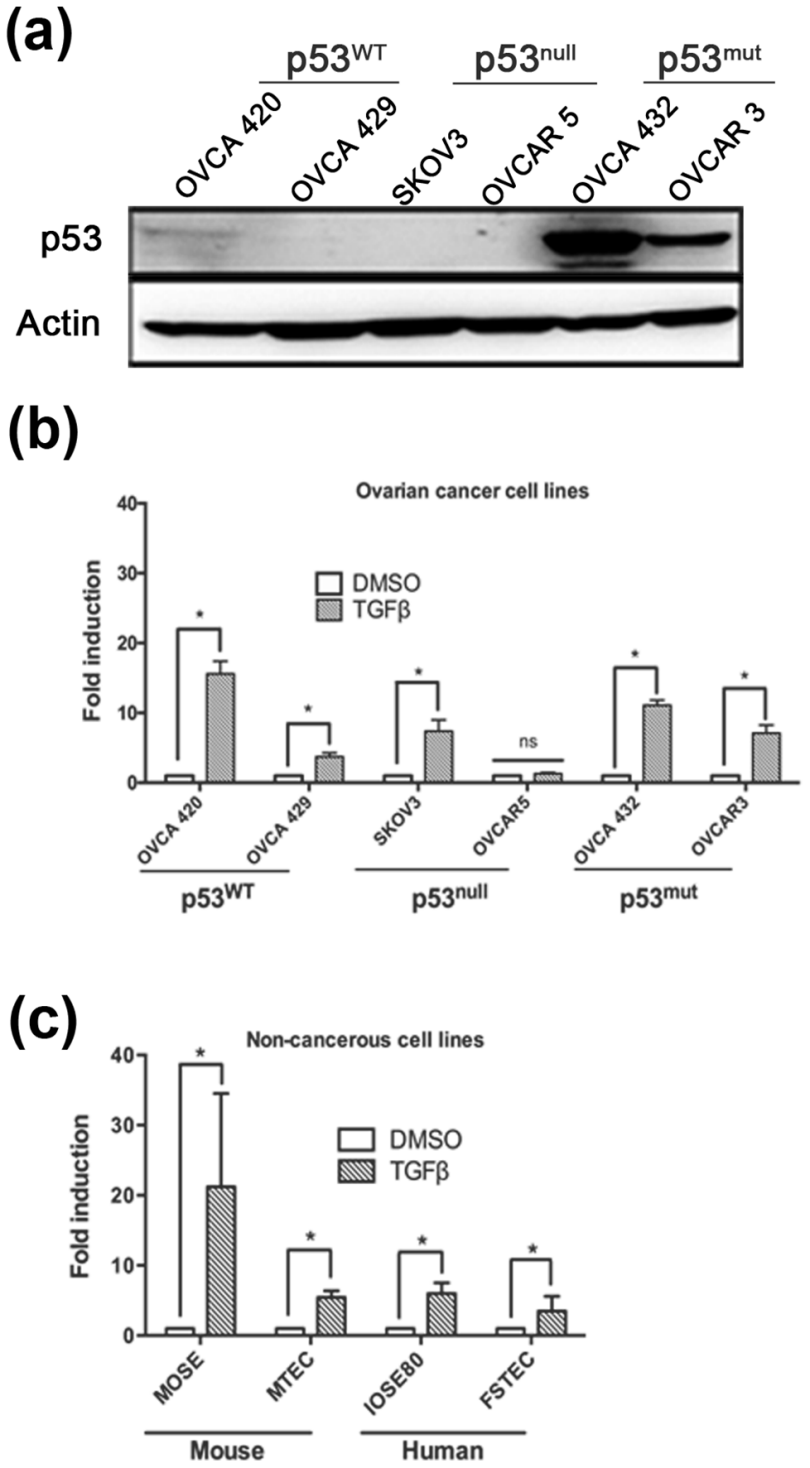
Ovarian cancer cell lines respond to TGFβ regardless of p53 status. (**a**) Western blot analysis of ovarian cancer cell lines demonstrating their p53 status. Actin used as a loading control. (**b**) Six ovarian cancer cell lines (OVCA 420, 429, SKOV3, OVCAR 5, OVCA 432, and OVCAR 3), along with four primary, non-cancerous cell lines (MOSE, MTEC, IOSE80, and FTSEC) were treated with or without TGFβ (10 ng/mL) using the SBE-luc plasmid. ANOVA was performed separately for fold induction (TGFβ) and fold repression (inhibitor and TGFβ + inhibitor) to analyze significance compared to untreated. Data represented as mean ± SEM, *p≤0.05.

To better understand the role of p53 in ovarian cancer, six known ovarian cancer cell lines were analyzed for p53 expression (**Fig. 1a**). OVCA 420 and 429 cells express low levels of p53 protein, consistent with reports that they have wild-type p53 [32]. Due to these low levels, cisplatin treatment was used to induce and confirm p53 expression in OVCA 429 (**Fig. S1**). SKOV3 and OVCAR5 did not show any p53 protein expression, as is consistent with the previous finding that classified them as p53 null [7]. In contrast, OVCA 432, and OVCAR3 exhibited abundant p53 protein expression due to the R277H and R248Q mutations, respectively (**Table S1**) [7].

To evaluate how p53 expression modulates the ability of cells to respond to Smad signaling, a luciferase assay was employed to determine which cells were responsive to TGFβ-induced Smad transcription (**Fig. 1b**). All cell lines tested, except OVCAR5, demonstrated TGFβ-mediated transcription that could be blocked by SB431542, a TGFβ inhibitor (data not shown).

Next, the effect of TGFβ on non-cancerous progenitor cells was investigated. Since ovarian surface epithelium (OSE) and fallopian tube epithelium (TEC) may give rise to ovarian cancer [33], the response of these normal cells to TGFβ was investigated. In order to monitor signaling downstream of TGFβ, SBE-luciferase assays were performed on normal 2D murine OSE (MOSE) and murine TEC (MTEC) cells, as well as human immortalized OSE (IOSE80) and human fallopian tube epithelium (FTSEC). OSE and TEC cells significantly responded to Smad-mediated transcription induced by TGFβ in both mouse and human cell lines (**Fig. 1b**). MOSE cells responded with a higher fold activation (21 fold) of the reporter than MTEC cells (7 fold). p53 expression in MOSE was previous confirmed as being wild-type [27], [34]. MTEC cells behaved as wild-type in response to cisplatin (**Fig. S2**), while the IOSE80 and FTSEC were functionally null for p53 due to immortalization with SV40.

**Figure 2:**
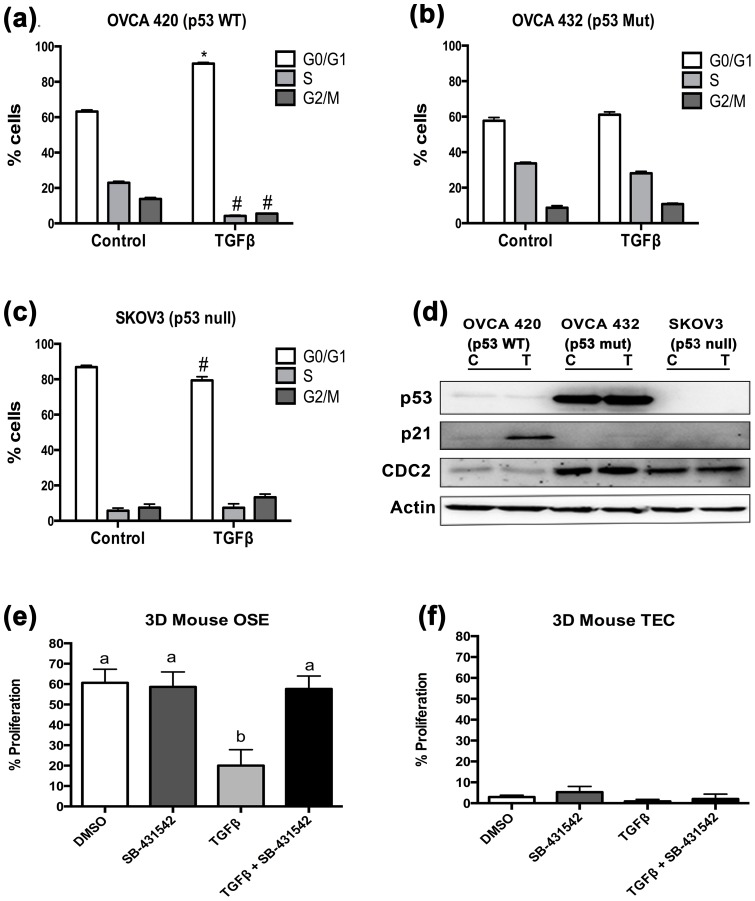
p53 wild-type ovarian cancer cell lines undergo cell cycle arrest in response to TGFβ. (**a–c**) OVCA 420, OVCA 432, and SKOV3 cell lines were treated with 20 ng/mL TGFβ for 24 hours and subjected to flow cytometry analysis. Distributions of cells in the three phases of the cell cycle are represented by mean percentages +/− SEM. Statistical significance represents a difference between number of cells in each cycle between treated and untreated and represented with * for an increase of treated cells compared to untreated; ^#^represents decrease of treated cells compared to untreated; p≤0.05 (**d**) Western blot analysis of the three ovarian cancer cell lines probed for cell cycle proteins p21 and CDC2. Actin was used as a loading control. (**e–f**) Proliferation assay performed using BrdU incorporation in 3D organ culture of mouse ovaries and tubes. One-way ANOVA was performed. Data represented as mean ± SEM *p≤0.05.

Based upon these results, three cell lines (OVCA 420, OVCA 432, and SKOV3) were chosen to further analyze the impact of TGFβ on proliferation. These cell lines were treated with TGFβ (20 ng/mL) for 48 hr and cell cycle progression was examined using flow cytometry. TGFβ treatment induced G_0_/G_1_ cell cycle arrest in OVCA 420 (**Fig. 2a**). OVCA 432 cells, which express mutant p53, were not growth arrested by TGFβ treatment (**Fig. 2b**). Lastly, TGFβ did not induce cell cycle arrest in SKOV3 cells, but rather reduced the number of cells in G_0_/G_1_ (**Fig. 2c**).

To further confirm the mechanism for the p53-Smad cell cycle regulation, expression of p21 and CDC2 were evaluated in OVCA 420, OVCA 432, and SKOV3 cells treated with TGFβ (**Fig. 2d**). p21 is a cyclin dependent kinase inhibitor that is regulated by both p53 and Smads, and correlates with TGFβ-mediated cell cycle arrest [4]. CDC2 (or CDK1) is a cyclin dependent kinase and its expression is consistent with cell cycle progression [35]. TGFβ treatment in OVCA 420 increased p21 protein expression (**Fig. 2a and d**), which was not observed in the OVCA 432 and SKOV3 cells (**Fig. 2d**). The OVCA 432 and SKOV3 cells expressed elevated levels of CDC2 as compared to OVCA 420 (**Fig. 2d**). Next, immunohistochemistry was performed to monitor proliferation of normal mouse ovarian surface epithelium and oviductal epithelium using a 3D organ culture system [30] treated with TGFβ. After 48 hr, OSE proliferation was significantly decreased with TGFβ treatment compared to DMSO control (**Fig. 2e**). Despite being transcriptionally responsive, proliferation was not inhibited by TGFβ treatment in oviductal cells in 3D culture (**Fig. 2f**). Immortalization of IOSE80 and FTSEC with SV40T antigen inactivates p53 [25], [26], therefore growth assays with TGFβ were not performed on these cells.

### TGFβ-induced cell cycle arrest is abrogated in p53 mutant and null cells

**Figure 3:**
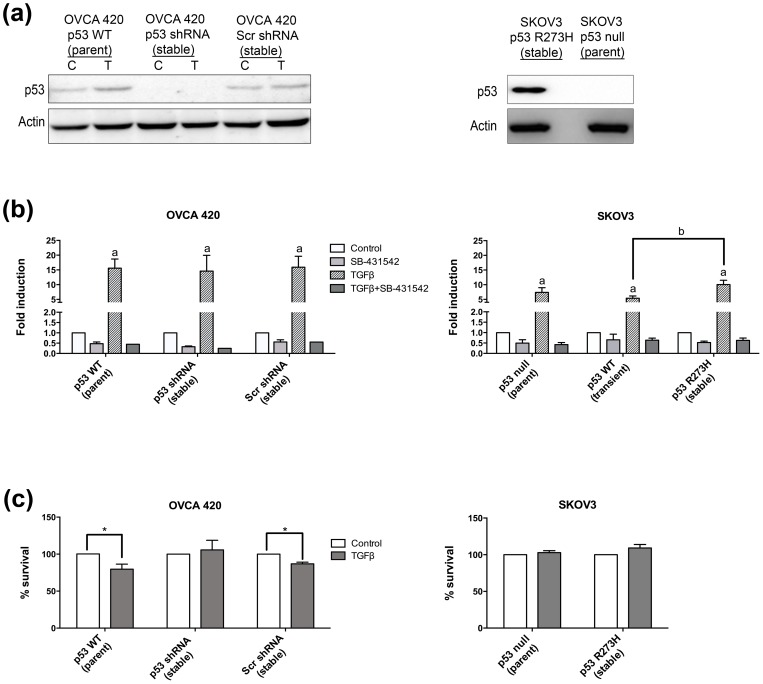
Wild-type p53 cells, but not p53 null or mutant p53 cells, are growth inhibited by TGFβ. (**a**) Western blot analysis of stable cell lines to knockdown of p53 by shRNA plasmid or expression of mutant p53 R273H. C = control, T = TGFβ treated. (**b**) SB-431542 (5 μM) was used to inhibit TGFβ signaling. For each panel, data represents mean ± SEM p≤0.05 increase over untreated for groups labeled with a, or between treated groups labeled with b. (**c**) Cell survival. Percentage of TGFβ-treated cell survival compared to untreated. Data represent mean ± SEM, *p≤0.05.

Variants of OVCA 420 were created to investigate the role of p53 in TGFβ-induced cell cycle arrest (Table S1 and S2). Using shRNA, endogenous wild-type p53 was effectively knocked down in OVCA 420 (OVCA 420 p53 shRNA) cells as compared to the scrambled shRNA control (OVCA 420 Scr) (**Fig. 3a**). Additionally, SKOV3 cells were stably transfected to express mutant p53 R273H (**Fig. 3a**). Wild-type p53 could not be stably transfected into SKOV3 cells because the cells underwent senescence and could not be propagated as previously reported [36]. Transient transfection of wild-type p53 into SKOV3 cells did not immediately induce senescence, which allowed data collection at shorter time points.

First, the ability of the variant cell lines to respond to TGFβ was investigated. All stable cell lines maintained the ability to induce Smad-mediated transcription of a SBE-luciferase plasmid irrespective of p53 status (**Fig. 3b**). Luciferase induction remained the same in the OVCA 420 p53 shRNA and OVCA 420 Scr when compared to the OVCA 420 parent cells (**Fig. 3b**). Similarly, the SKOV3 stable mutant p53 R273H cells did not alter TGFβ-induced Smad-mediated transcription of the luciferase gene (**Fig. 3b**). Transient expression of wild-type p53 in the SKOV3 cells reduced Smad-mediated transcription in comparison to the SKOV3 mutant p53 R273H cells, but displayed no significant difference compared to the SKOV3 parent cell line (**Fig. 3b**).

In order to assess proliferation in response to TGFβ (20 ng/mL), cell growth assays were performed after 48 hr incubation. As expected, OVCA 420 Scr cell growth was repressed in response to TGFβ, which was similar to the parental line (**Fig. 3c**). TGFβ induced growth inhibition was lost in the OVCA 420 p53 shRNA. Similarly, TGFβ did not slow proliferation in either the parent SKOV3 or the SKOV3 mutant p53 R273H cell line (**Fig. 3c**).

### Mutant p53 R273H expression prevents TGFβ-induced migration of SKOV3 cells

**Figure 4:**
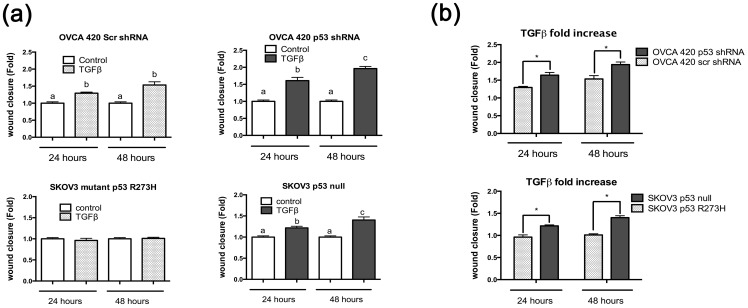
TGFβ induces migration in p53 null cells in comparison to p53 wild-type or mutant cells. (**a**) Wound healing assays were performed on SKOV3 and OVCA 420 stable cell lines. Cell monolayers were scratched and treated with or without TGFβ at 20 ng/mL for 48 hours. Wound closure was measured as a fold increase or decrease compared to no treatment control. Paired t-test was used with a p≤0.05. (**b**) Comparison of the fold increase of TGFβ samples from 5(a). Unpaired t-test was used to analyze significance. Significance is represented by * and signifies a statistical difference between cell lines. Data represented as mean ± SEM, *p≤0.05.

In addition to affecting proliferation, TGFβ and p53 have also been shown to influence migration of tumor cells from the breast and lung [8], [9], [37]. Previous literature suggests that mutant p53 might function as a molecular trigger allowing TGFβ to induce pro-migratory stimuli [38]. Therefore, TGFβ regulation of cell migration in the presence of wild-type, mutant, and null p53 was investigated using a wound healing assay. TGFβ induced migration in both OVCA 420 Scr and OVCA 420 p53 shRNA cells between 0 and 24 hours and 24 and 48 hours (**Fig. 4a**). OVCA 420 Scr (p53 wild-type) cells treated with TGFβ migrated significantly more than non-treated control between 0 and 24 hours, but not between 24 hours and 48 hours, whereas OVCA 420 p53 shRNA cells treated with TGFβ migrated significantly more than control between 0 and 24 hours and between 24 and 48 hours (**Fig. 4a**). Migratory rates were compared between treated OVCA 420 Scr and OVCA p53 shRNA. Knockdown of p53 allowed for an increased migration compared to wild-type cells (**Fig. 4b**).

The ability of SKOV3 null and SKOV3 mutant p53 R273H cells to migrate in response to TGFβ was also analyzed. TGFβ induced migration in p53 null SKOV3 cells compared to untreated cells between both 0 and 24 hours and between 24 and 48 hours (**Fig. 4a** **)**. However, expression of mutant p53 R273H in SKOV3 cells inhibited TGFβ-induced migration, with no change between 0 and 24 hours, or 24 and 48 hours when compared to control (**Fig. 4a**). SKOV3 cells expressing mutant p53 R273H demonstrated less TGFβ-induced migration than SKOV3 null cells (**Figure 4b**).

### Expression of mutant p53 R273H alters TGFβ induced-expression of TMEPAI and DKK1

**Figure 5:**
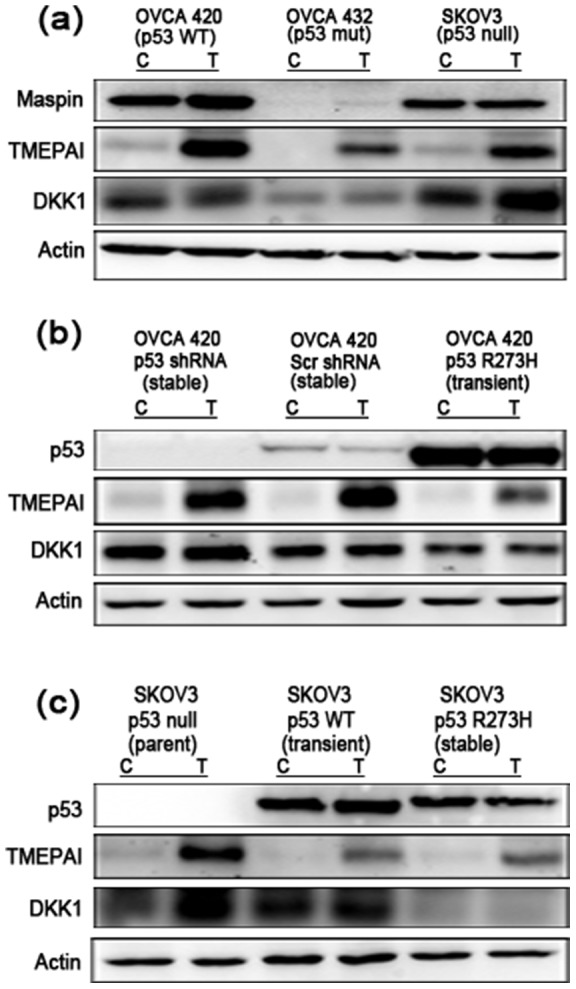
TGFβ-induced expression of pro-metastatic proteins is upregulated in p53 null cells. (**a**) OVCA 420 (p53 wild-type), OVCA 432 (p53 mutant), and SKOV3 (null p53) cells were treated with 10 ng/mL TGFβ for 24 hours and analyzed by western blotting. Membranes were probed with Maspin, TMEPAI and DKK1 primary antibodies. Actin was used as an internal loading control. (**b**) OVCA 420 cell lines were analyzed by western blot and probed for pro-metastatic factors TMEPAI, and DKK1. Actin was used as an internal loading control. (**c**) SKOV3 cell lines were analyzed by western blot and probed for pro-metastatic factors TMEPAI and DKK1. SKOV3 p53 WT was transiently transfected with 100 ng/mL of p53 wild-type plasmid. Actin was used as an internal loading control.

In order to elucidate possible mechanisms by which p53 and TGFβ might regulate migration, pro-invasive targets known to be regulated by either p53 or TGFβ in ovarian cancer cells were investigated. Maspin is a serine protease inhibitor that blocks metastasis [20] and is known to be co-regulated by p53 and Smads in mammary epithelial cells [20]. Additionally, maspin expression is reportedly lost in ovarian cancers, and this has been associated with poor prognosis and survival rates [39]. Maspin was minimally induced with TGFβ treatment in OVCA420 and OVCA432 cells (**Fig. 5a**), and was not induced in SKOV3. Surprisingly, maspin did not demonstrate a dependence on TGFβ treatment or p53 expression in OVCA420 p53 shRNA or SKOV3 R273H mutant p53 cell lines (data not shown) compared to parent cells suggesting that additional pathways modify p53 and Smad regulation of maspin in ovarian cancer cells.

TMEPAI is a TGFβ-induced protein that is known to convert TGFβ from a tumor suppressor into a tumor promoter in breast cancer, and is associated with increased migration in prostate and renal carcinomas [18], [40], [41]. Overexpression of TMEPAI has been associated with many cancers, including ovarian cancer [42]. TGFβ increased expression of TMEPAI in p53 wild-type OVCA 420 cells and null SKOV3 cells (**Fig. 5a**). Mutant R277H p53 OVCA 432 cells demonstrated a reduced induction of TMEPAI expression. TGFβ-induced TMEPAI expression in OVCA 420 mutant p53 R273H transient cells was reduced in comparison to wild-type and null p53 cells (**Fig. 5b**). Similarly, TMEPAI induction by TGFβ in SKOV3 mutant p53 R273H cells was lower than that of SKOV3 wild-type and null p53 cells (**Fig. 5c**).

Lastly DKK1, a Wnt-signaling inhibitor, was selected as it is differentially regulated by wild-type and mutant p53, and is also overexpressed in late stage metastatic ovarian cancers [19]. TGFβ induced expression of DKK1 in SKOV3 null p53 cells (**Fig. 5a**). This increase was not seen in wild-type OVCA 420 or mutant R277H p53 OVCA 432 cells. In OVCA 420 cells, overall levels of DKK1 were highest in OVCA 420 p53 shRNA, with a slight induction upon TGFβ treatment (**Fig. 5b**). OVCA 420 mutant p53 R273H had the lowest amount of DKK1, with no induction upon TGFβ treatment (**Fig. 5b**). In SKOV3 cells, the parent cell line (null p53), displayed higher DKK1 protein after TGFβ treatment as compared to the transiently transfected wild-type and mutant p53 R273H SKOV3 cells (**Fig. 5c**).

## Discussion

This study investigated the influence of p53 on TGFβ-mediated proliferation and migration in ovarian cancer. Expression of p53 did not alter the ability of the ovarian cancer cells to respond to TGFβ. However, the p53 status did affect proliferation, migration, and expression of pro-invasive genes. Wild-type p53 cells underwent cell cycle arrest and displayed an inhibition of proliferation when treated with TGFβ. Loss or expression of mutant p53 abrogated TGFβ growth arrest. Interestingly, while both the OSE and TEC responded transcriptionally to TGFβ, only mouse OSE were growth inhibited by TGFβ, indicating a unique action in different potential progenitor cells. Stable integration of mutant p53 R273H mitigated TGFβ-induced migration. In correlation with these functional data, *TMEPAI* and *DKK1* were most significantly upregulated by TGFβ in null and wild-type p53 cells, while expression of these proteins was lower in cells expressing mutant p53 R273H. Therefore, although mutant and null p53 ovarian cancer cells are not growth inhibited by TGFβ, the loss of p53 enhances migration and pro-migratory gene expression induced by TGFβ more than mutation of p53.

In breast and lung cancers, mutant p53 interacts with Smads to alter transcription of genes that regulate metastasis, but little is known about how p53 and TGFβ interact in ovarian cancer. Mutant p53 R273H is the most common p53 mutation in ovarian cancer [7], but recent evidence suggests that silencing or null mutations in p53 may be more metastatic in ovarian cancer, and that mutant p53 retains some wild-type activity [43], [44]. Additionally, analyses of gene signatures from metastatic serous ovarian cancers highlighted TGFβ's involvement in the metastatic disease [45]. While breast and colon cancers undergo intra- and extravasation in order to metastasize, ovarian cancer metastasizes through direct dissemination into the peritoneal cavity [17]. Therefore, signal transduction necessary for metastasis in breast, lung, and colon cancer may be different in ovarian cancer, leading to tissue specific effects of mutant p53 and TGFβ signaling [17].

The use of TGFβ inhibitors in the treatment of ovarian cancer has been explored and is dependent on many factors [46]. Based on the current study, in high-grade serous cancers, if p53 activity is lost, TGFβ inhibitors may provide greater therapeutic value than in mutant p53 R273H tumors. TGFβ inhibitors may also have a differential impact on cancers arising from the OSE or TEC, the two potential cell types of origin. While both the OSE and TEC in mouse and human cell lines respond transcriptionally to TGFβ, the OSE may respond more robustly than the TEC. In addition, MOSE were growth inhibited by TGFβ treatment, while the MTEC were not. Previous data demonstrated ovarian cancer cell metastasis is reduced in response to TGFβ inhibitors; however, the study did not control for the p53 status of the cells grafted and did not identify the cell of origin [46], [47]. Several TGFβ inhibitors are currently in pre-clinical and clinical trials as cancer therapeutics [48].

TMEPAI and DKK1 induction by TGFβ was lower in ovarian cancer cells containing mutant p53 R273H, which were also less migratory. TMEPAI is associated with metastatic disease [18] and has been reported to enhance TGFβ-induced migration and an epithelial-to-mesenchymal transition (EMT) in early and late stage tumors of the breast and lung [18], [49]. Additionally, the chromosomal region containing *TMEPAI* has been reported to be duplicated in breast and ovarian cancer [40]. DKK1 expression is often upregulated in ovarian cancer and has been associated with poor outcome [19]. Although the exact role of DKK1 in the aggressive nature of ovarian cancer is unknown, it has been proposed as a useful marker of disease and may be one of the many factors contributing to high-grade serous ovarian cancer [19]. DKK1 induction by TGFβ was higher in cells that lacked p53 when compared to cells with mutant p53 and these cells also displayed the highest level of TGFβ-induced migration.

The early initiation and progression mechanisms of ovarian cancer are not well understood due to the poor early detection strategies, leading to a deadly, highly aggressive disease. These data provide some insight into the role of mutant p53 in ovarian cancer and how it intersects with the TGFβ signaling pathway. In ovarian cancer cell lines with mutated or null p53, growth inhibition from TGFβ is lost. Mutant p53 R273H did not induce the same pro-migratory function in response to TGFβ in ovarian cancer as it had in breast and lung cancer cells [9], [37]. In addition, TGFβ inhibited the growth of normal mouse OSE, but not mouse TEC. These findings suggest that the p53 status of ovarian cancer cells influences their proliferative and migratory behavior when exposed to TGFβ.

## Supporting Information

Figure S1 **Induction of p53 in OVCA 429 wild-type p53 cells by cisplatin challenge.** Cells were treated with 125 μM of cisplatin for 2 hours and cell lysates run on a western blot. Actin was used as an internal loading control.
(TIF)

Figure S2 **p53 Status in MTEC cells.** Cells were treated with 125 μM of cisplatin for 2 hours and cell lysates run on a western blot. Actin was used as an internal loading control.
(TIF)

Table S1 **Ovarian cancer cell lines and their p53 status.**
(DOCX)

Table S2 **Parent cell line variants and their manipulations.**
(DOCX)
