# Supplementary figures and images for: Mutation or Loss of p53 Differentially Modifies TGFβ Action in Ovarian Cancer
Source: PLoS One. 2014 Feb 20;9(2):e89553. doi: 10.1371/journal.pone.0089553 (PMC3930740; doi:10.1371/journal.pone.0089553)

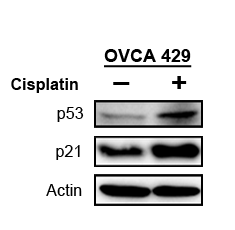

Supplement: Figure S1 — Induction of p53 in OVCA 429 wild-type p53 cells by cisplatin challenge. Cells were treated with 125 μM of cisplatin for 2 hours and cell lysates run on a western blot. Actin was used as an internal loading control. (TIF) [file pone.0089553.s001.tif]

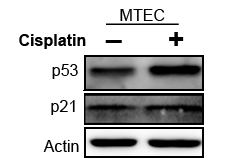

Supplement: Figure S2 — p53 Status in MTEC cells. Cells were treated with 125 μM of cisplatin for 2 hours and cell lysates run on a western blot. Actin was used as an internal loading control. (TIF) [file pone.0089553.s002.tif]
