# Supplementary material for: Mutation or Loss of p53 Differentially Modifies TGFβ Action in Ovarian Cancer
Source: PLoS One. 2014 Feb 20;9(2):e89553. doi: 10.1371/journal.pone.0089553 (PMC3930740; doi:10.1371/journal.pone.0089553)
Supplement: Table S1 — Ovarian cancer cell lines and their p53 status. (DOCX) [file pone.0089553.s003.docx]

**Table S1: Parent cell lines and their p53 status**

| Parent cell lines | p53 Wild-type (WT) | p53 absent (null) | p53 mutation (MT) | Reference |
| --- | --- | --- | --- | --- |
| OVCA420 | X |  |  | [21,22,32] |
| OVCA429 | X |  |  | [21,22,32] |
| SKOV3 |  | X |  | [7] |
| OVCAR5 |  | X |  | [7] |
| OVCA432 |  |  | X (R277H) | [7,21,22] |
| OVCAR3 |  |  | X (R248W) | [7] |
| IOSE80 |  | X |  | [25] |
| FTSEC |  | X |  | [26] |
