# Supplementary material for: Mutation or Loss of p53 Differentially Modifies TGFβ Action in Ovarian Cancer
Source: PLoS One. 2014 Feb 20;9(2):e89553. doi: 10.1371/journal.pone.0089553 (PMC3930740; doi:10.1371/journal.pone.0089553)
Supplement: Table S2 — Parent cell line variants and their manipulations. (DOCX) [file pone.0089553.s004.docx]

**Table S2: Parent cell variants and their manipulation**

| Modified (variant) cell lines | Stable | Transient |
| --- | --- | --- |
| OVCA420 p53 shRNA | X |  |
| OVCA420 scrambled shRNA (Scr) | X |  |
| OVCA420 p53 R273H |  | X |
| SKOV3 p53 Wild-type (WT) |  | X |
| SKOV3 p53 R273H | X |  |
